# Out-of-State Acute Care Use Among Pediatric Medicaid Enrollees

**DOI:** 10.1001/jamanetworkopen.2025.36236

**Published:** 2025-10-07

**Authors:** Kenneth A. Michelson, Naveen Singamsetty, Andrew D. Skol, Katherine E. Remick, Emily M. Bucholz, John A. Graves, Danielle K. Cory, Patrick D. McMullen

**Affiliations:** 1 Division of Emergency Medicine, Ann & Robert Lurie Children’s Hospital, Chicago, Illinois; 2 Stanley Manne Children’s Research Institute, Ann & Robert Lurie Children’s Hospital, Chicago, Illinois; 3 Department of Pediatrics, Dell Medical School, University of Texas at Austin, Austin; 4 Section of Cardiology, Department of Pediatrics, University of Colorado and Children’s Hospital Colorado, Aurora; 5 Department of Health Policy, Vanderbilt University School of Medicine, Nashville, Tennessee

## Abstract

**Importance:**

For many US children, the nearest hospital may be out of state. Medicaid coverage differs by state, affecting access across state lines.

**Objective:**

To evaluate the frequency of out-of-state acute care use for pediatric patients.

**Design, Setting, and Participants:**

This cross-sectional study analyzed acute care hospital data for emergent and inpatient encounters among children younger than 16 years enrolled in Medicaid or the Children’s Health Insurance Program (CHIP) in the 2021-2022 Transformed Medicaid Statistical Information System Analytic File database. Analyses were conducted January to July 2025.

**Exposure:**

Distance from a state border.

**Main Outcomes and Measures:**

The primary outcome was out-of-state care. The percentages of encounters occurring out of state were measured by state, city, and zip code. Logistic regression was used to evaluate the association of out-of-state care use with the log distance from a patient’s zip code to the border between states.

**Results:**

This analysis included 28 952 692 acute care patient encounters (median [IQR] age, 5.3 [2.0-10.8] years, 52.3% male). Out-of-state care occurred among 820 972 encounters (2.8% [95% CI, 2.8%-2.8%]). Maryland (61 468 of 389 539 [15.8% (95% CI, 15.7%-15.9%)]), Vermont (3625 of 31 101 [11.7% (95% CI, 11.3%-12.0%)]), and West Virginia (18 455 of 168 151 [11.0% (95% CI, 10.8%-11.1%)]) had the highest percentages of out-of-state care. The city from which the highest number of children accessed care out of state was Kansas City, Missouri (13 327 of 84 181 encounters [15.8% (95% CI, 15.6%-16.1%)]). Out-of-state care use was more common in rural areas (4.4% [95% CI, 4.3%-4.4%]) compared with urban areas (2.7% [95% CI, 2.7,%-2.7%]). For every 2-fold increase in distance from a state border, crossing a border for care was 34.2% (95% CI, 34.2%-34.3%) less likely. Among children within 1 mile of a state border, 10.0% (95% CI, 9.9%-10.0%) received care out of state.

**Conclusions and Relevance:**

Findings from this cross-sectional study of Medicaid and CHIP enrollees indicated that out-of-state acute care use was uncommon overall but more common near state borders. Certain states and cities had high rates of out-of-state acute care use. Changes to Medicaid reimbursement could affect patients’ ability to access cross-border care.

## Key Points

**Question:**

How often do US children cross state borders for acute care?

**Findings:**

This cross-sectional study found that for 28.9 million encounters involving children younger than 16 years enrolled in Medicaid or the Children’s Health Insurance Program, 2.8% of all acute care encounters occurred out of state, with 10.0% living within a mile of a border crossing state lines for care.

**Meaning:**

These findings suggest that publicly insured children access out-of-state acute care infrequently, but for those who do, changes in insurance coverage between states may alter their access to care.

## Teaser

This cross-sectional study analyzing data from more than 28 million children enrolled in Medicaid or the Children’s Health Insurance Program evaluates the frequency of out-of-state acute care use.

## Introduction

Many families weigh different options in where they seek acute care for their children. Most children arrive at the emergency department (ED) via private vehicle, and a third to half bypass their nearest hospital.^1,2,3^ Reasons given to bypass the nearest hospital include differences in capabilities or personal preference.^2,3,4,5^ For many, the most capable or specialized hospital may be out of state.

The frequency and determinants of out-of-state acute care are important for policy and research. This is particularly true for children who receive public insurance coverage, either from Medicaid or the Children’s Health Insurance Program (CHIP), who represented 59.1% of all US children in 2021 (the year corresponding to our data).^6,7,8^

From a policy perspective, cross-state care poses unique challenges. Although Medicaid technically covers out-of-state care, it is often reimbursed at lower rates than in-state care, which may make health care professionals less likely to accept out-of-state patients.^9^ Furthermore, administrative hurdles, including credentialing, billing, prior authorizations, legal ramifications (eg, in assault or abuse cases that may create cross-state criminal justice issues), and care coordination across state lines, create additional disincentives for hospitals to accept out-of-state Medicaid patients. Thus, understanding which children regularly receive out-of-state care is essential for informing Medicaid reimbursement policy and improving access to care for pediatric patients.

From a research perspective, many pediatric health services studies rely on state-specific administrative datasets, such as Medicaid claims or hospital discharge databases.^10^ Yet these databases often fail to capture out-of-state encounters, leading to missing data and the potential for systematic underestimation of care use for border populations. This may lead to biased estimates of access and quality or obscure the role of regional referral patterns.

Despite these implications, the national frequency and geographic distribution of out-of-state pediatric care remain poorly understood. To address this gap, we used a national public payer database representing 59% of US children to (1) determine the prevalence and variation in out-of-state acute care use for pediatric encounters, (2) evaluate the association between a patient’s distance to a state border and out-of-state care, and (3) examine the extent to which specific border cities are associated with out-of-state pediatric acute care nationally.

## Methods

This was a descriptive cross-sectional study of pediatric acute care encounters. The source for encounter data was the 2021-2022 Transformed Medicaid Statistical Information System Analytic File (TAF), an administrative database containing all claims for Medicaid and CHIP for the entire US, comprising 59% of all US children in 2021.^6,7,8^ The source for hospital data was the National Plan and Provider Enumeration System (NPPES). TAF and NPPES were linked using hospitals’ National Provider Identifier, which uniquely identifies hospitals and other provider types. This study was considered exempt from review and informed consent was waived by the Ann & Robert Lurie Children's Hospital Institutional Review Board, Chicago, Illinois, because patients were anonymized and could not be identified. We followed the Strengthening the Reporting of Observational Studies in Epidemiology (STROBE) reporting guideline in preparing this manuscript.^11^

Acute care encounters among patients under 16 years of age in any of the 50 US states and the District of Columbia, Puerto Rico, and the US Virgin Islands (hereafter all referred to as states for brevity) were eligible. We used a cutoff of 16 years of age because it most specifically defines the pediatric cohort.^12^ We defined acute care as inpatient (identified in the TAF inpatient claims file) or ED encounters (from TAF other services file facility claims with a revenue center code of 0450-0459, 0762, or 0981).^13^ We excluded hospitals not providing substantial acute pediatric care (defined as <52 encounters during the 2-year study period or those without an NPPES taxonomy code for acute care hospitals), encounters with missing zip code, birth hospitalizations (which are distinct from acute care), and psychiatric encounters (which have distinct use patterns).

### Outcomes

The outcome was out-of-state acute care use, defined as a mismatch between the patient’s home state (based on zip code) and the hospital’s state. We assigned zip codes to states using the 2021 US Department of Housing and Urban Development crosswalk.^14^ We obtained hospitals’ state locations from NPPES using the actual place of service address. For the top 50 hospitals receiving out-of-state patients, we verified the addresses using Google search, correcting 1 NPPES state.

### Variables

We obtained demographics for patients at the time of the encounter, including age and sex, as recorded in the database. We determined zip code cities from US Housing and Urban Development assignments.^14^ Zip urban or rural status (urban, micropolitan, or rural) was based on the Census Bureau Core-Based Statistical Area definitions. We measured the distance from each zip code to a state border based on the shortest straight line from any point in the zip code tabulation area to any state border. Hospitals were considered pediatric hospitals if they self-identified as such in NPPES and otherwise were considered general hospitals. For each zip code, we determined the nearest hospital and nearest children’s hospital based on straight-line distances from each zip code centroid.

### Statistical Analysis

We first determined the percentage of encounters and patients seeking out-of-state acute care in the US. We then mapped the percentages of encounters occurring out of state by zip code. We hypothesized that pediatric hospitals have a wider catchment than general hospitals. Thus, we determined the percentage of encounters out of state for children’s and general hospitals.

We examined the association between patient distance from a state border and out-of-state care use. We determined percentages of out-of-state encounters at different distance thresholds. We created a logistic regression model with out-of-state care as the outcome and log distance from state border as the sole covariate. We chose a logarithmic association because it best fit the association between distance and out-of-state care based on the Akaike information criterion, compared with a linear or square root association. We then constructed a multivariable model by adding binary indicators for whether the nearest hospital was out of state, whether the nearest children’s hospital was out of state, and their interaction term.

We hypothesized that a larger proportion of out-of-state care use would occur in cities that straddle or are near state borders. To evaluate this, we first determined the percentage of cities from which less than 1% of encounters occurred out of state, and at thresholds of more than 50% and more than 90% of encounters out of state. We then determined which 5 cities had the most encounters occurring out of state. For the city with the highest number, we mapped the flow from each zip code within city limits.

We reported 95% CIs using the binomial exact method. We used R, version 4.4.0 (R Project for Statistical Computing), for analyses and the sf package for maps. Analyses were conducted January to July 2025.

## Results

Among 33.0 million acute care encounters, we excluded 0.6 million (1.9%) that occurred in a nonacute care hospital, 0.3 million (1.0%) with missing zip codes, 0.2 million (0.5%) with psychiatric encounters, and 2.9 million (8.9%) for birth hospitalizations. We thus analyzed 28.9 million (28 952 692 [87.7%]) encounters (patient median [IQR] age, 5.3 [2.0-10.8] years; 13.8 million [47.7%] female, and 15.1 million [52.3%] male) arising from 14.0 million unique patients in 4776 hospitals. Other demographic characteristics of visits and hospitals are shown in the Table.

**Table.** Demographic Characteristics of Patient Encounters and Hospitals

Characteristic	Encounters or hospitals, No. (%)
**Encounters**	
No.	28 952 692
Age, median (IQR), y	5.3 (2.0-10.8)
Sex, millions	
Female	13.8 (47.7)
Male	15.1 (52.3)
Missing	<0.1 (0.0)
Urban or rural status, millions	
Metropolitan	24.2 (83.6)
Micropolitan	2.8 (9.7)
Rural	1.9 (6.7)
Missing	<0.1 (0.0)
Encounter type, millions	
Emergency department	27.3 (94.2)
Hospitalization	1.7 (5.8)
Missing	0
**Hospitals**	
No.	4776
Urban or rural status	
Metropolitan	2937 (61.5)
Micropolitan	770 (16.1)
Rural	1069 (22.4)
Hospital type	
Children’s	4650 (97.4)
Nonchildren’s	126 (2.6)
Pediatric Medicaid or CHIP volume, median (IQR), No./y	1370 (538-2980)

Abbreviation: CHIP, Children’s Health Insurance Program.

Children received care out of state in 820 972 encounters nationally (2.8% [95% CI, 2.8%-2.8%]). Among unique patients, 7 434 048 (53.3% [95% CI, 53.3%-53.3%]) had 1 visit, 3 160 807 (22.7% [95% CI, 22.6%-22.7%]) had 2 visits, and 3 355 710 (24.1% [95% CI, 24.0%-24.1%]) had 3 or more visits. Among 6 516 517 patients who had at least 2 visits, 6 220 027 (95.5% [95% CI, 95.4%-95.5%]) occurred exclusively in state, 100 951 (1.5% [95% CI, 1.5%-1.6%]) occurred exclusively out of state, and 195 539 (3.0% [95% CI, 3.0%-3.0%]) occurred in a combination of in- and out-of-state facilities.

**Figure 1.**
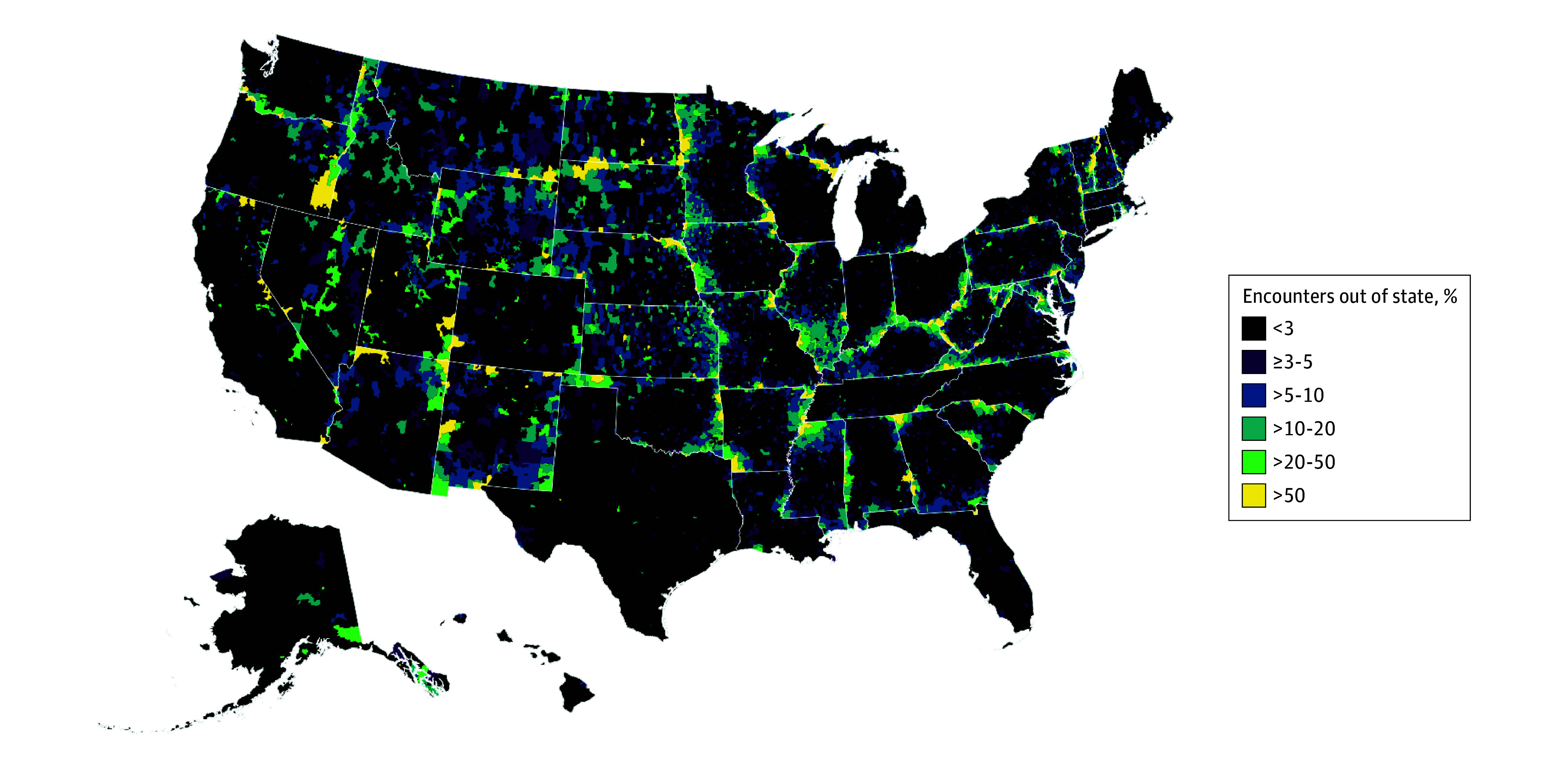
Percentage of Pediatric Acute Care Encounters Occurring Out of State From Each US Zip Code

Percentages of encounters occurring out of the patient’s home state by zip code are shown in Figure 1. Across states, the median (IQR) number of acute care encounters was 389 539 (92 684-694 469). The median (IQR) number of out-of-state encounters across states was 13 378 (3625-21 779). The states with the highest rates of out-of-state care encounters were Maryland (61 468 of 389 539 [15.8% (95% CI, 15.7%-15.9%)]), Vermont (3625 of 31 101 [11.7% (95% CI, 11.3%-12.0%)]), and West Virginia (18 455 of 168 151 [11.0% (95% CI, 10.8%-11.1%)]). The states with the lowest rates of out-of-state care encounters were Puerto Rico (1467 of 262 414 [0.6% (95% CI, 0.5%-0.6%)]), California (20 470 of 2 863 881 [0.7% (95% CI, 0.7%-0.7%)]), and Texas (24 464 of 3 235 364 [0.8% (95% CI, 0.7%-0.8%)]) (eTable in Supplement 1). Children’s hospitals’ encounters originated from out of state in 264 611 of 5 243 450 cases (5.0% [95% CI, 5.0%-5.1%]), whereas general hospitals’ encounters originated from out of state in 556 361 of 23 704 566 cases (2.3% [95% CI, 2.3%-2.4%]). Encounters among urban patients were less likely (644 888 of 24 194 247 [2.7% (95% CI, 2.7%-2.7%)]) to occur out of state than those among micropolitan (91 120 of 2 808 916 [3.2% (95% CI, 3.2%-3.3%)]) or rural (84 951 of 1 944 692 [4.4% (95% CI, 4.3%-4.4%)]) patients.

**Figure 2.**
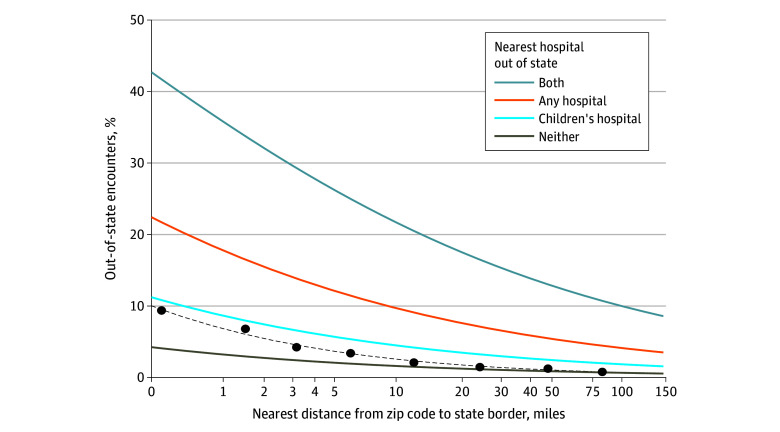
Modeled Associations Between Zip Code Distance From a State Border and the Percentage of Pediatric Encounters Occurring Across a State Line Unadjusted associations are shown as a dashed line, with raw percentages shown as points calculated in groups of 0 to less than 1 mile, 1 mile to less than 2 miles, and including all groups with ranges doubling until 64 miles to less than 128 miles. Adjusted associations are shown by whether the nearest hospital and nearest children’s hospital to a patient’s zip code were out of state.

Encounters among children living close to a state border were more likely to occur out of state. Among children residing in zip codes 1 mile or less from a border, 329 203 of 3 303 724 encounters (10.0% [95% CI, 9.9%-10.0%]) were out of state; from 1 to 5 miles, 149 120 of 3 204 171 encounters (4.7% [95% CI, 4.6%-4.7%]); from 5 to 10 miles, 88 124 of 3 020 959 encounters (2.9% [95% CI, 2.9%-2.9%]); from 10 to 20 miles, 105 343 of 6 089 978 encounters (1.7% [95% CI, 1.7%-1.7%]); from 20 to 50 miles, 78 262 of 6 152 236 encounters (1.3% [95% CI, 1.3%-1.3%]); and 50 or more miles, 70 898 of 7 175 273 (1.0% [95% CI, 1.0%-1.0%]). For every 2-fold increase in distance, encounters were 34.2% (95% CI, 34.2%-34.3%) less likely to occur out of state (Figure 2). In the multivariable model, after adjustment for whether the nearest hospital or nearest children’s hospital was out of state, every 2-fold increase in distance was associated with a 25.1% (95% CI, 25.0%-25.2%) decrease in likelihood of out-of-state care use.

**Figure 3.**
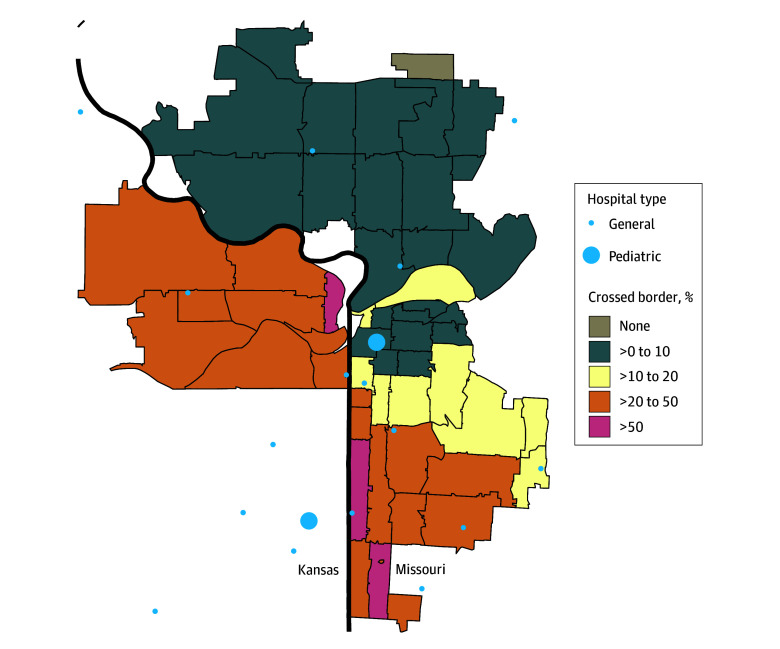
Percentage of Pediatric Patients Crossing the Kansas-Missouri Border by Kansas City Zip Code and Locations of Hospitals Heavy black line indicates the border. Out-of-city zip codes are not shown.

Among 27 313 cities, 12 868 (47.1% [95% CI, 46.5%-47.7%]) had less than 1% of residents’ encounters occurring out of state, 635 (2.3% [95% CI, 2.1%-2.5%]) had more than 50% occurring out of state, and 127 (0.5% [95% CI, 0.4%-0.6%]) had more than 90% occurring out of state. The cities with the highest out-of-state care use were Kansas City, Missouri (13 327 of 84 181 encounters [15.8% (95% CI, 15.6%-16.1%)]), Kansas City, Kansas (9983 of 25 142 encounters [39.7% (95% CI, 39.1%-40.3%)]), Hyattsville, Maryland (9630 of 14 858 encounters [64.8% (95% CI, 64.0%-65.6%)]), Bronx, New York (6774 of 273 816 encounters [2.5% (95% CI, 2.4%-2.5%)]), and Vancouver, Washington (6456 of 21 865 encounters [29.5% (95% CI, 28.9%-30.1%)]). Kansas City, Missouri, and Kansas City, Kansas, appeared to have a frequent flow of patients across their state borders (Figure 3).

## Discussion

This descriptive cross-sectional study found that most children in the US received acute care in state, but those living near a state border often sought care out of state. This finding varied considerably from region to region. Certain states, such as Maryland (with residents leaving), and certain cities, such as Kansas City, Missouri, and Kansas City, Kansas, had frequent cross-border acute care use. Living near a border was a key characteristic for seeking out-of-state care, even after adjustment for whether the nearest hospital was out of state, suggesting that border communities may rely on the ability to cross state lines for acute care. These findings have implications both for Medicaid policy and health services research.

Single-state databases are popular for health services research as they are the only available all-payer source of administrative data currently. Examples include the Healthcare Cost and Utilization Project state databases^15^ and state-specific all payer claims databases.^16^ When patients are followed up longitudinally between care sites, there is a high risk of missing care when families cross borders. Our data indicated that this was most likely in zip codes touching or close to state borders.

This study has several policy implications. Providers—facilities and individual health care professionals—are not obliged to accept out-of-state insurance. The choice of doing so depends heavily on specific plan benefits. For example, neurosurgeons and office-based physicians are more likely to accept Medicaid plans with higher reimbursement relative to Medicare.^17,18^ Pediatric specialists are less likely to accept Medicaid and offer longer wait times to see Medicaid-enrolled children, suggesting that reimbursement drives access.^19^ When Medicaid is not accepted from out-of-state patients, hospitals are more likely to be financially vulnerable because of uncompensated care, and families are more likely to face catastrophic bills or forgo care.^20,21^ Currently, particularly in Medicaid expansion states, hospitals do commonly accept out-of-state plans.^9^ However, with Medicaid threatened by the current federal spending legislation, this reciprocity may be tested in the future.^22^

Medicaid managed care arrangements cover 70% of Medicaid enrollees.^23^ At present, federal regulations^24^ require interstate cooperation and specify that plans must cover emergency care, care when travel would present harms, cross-border care that is common in the community, or when resources are only practicably available across the border. Reimbursement cuts could incentivize states to advocate rolling back these protections to limit payments to hospitals in other states. In addition, managed care plans could push patients toward lower-cost, in-state options. Given the already high rates of claim denials in Medicaid managed care,^21^ future work should evaluate whether claim denial rates are higher out of state compared with in state. States may also legislate how hospitals and plans handle out-of-state care, increasing protections for patients.

### Limitations

This study has limitations. First, we included only children with Medicaid or CHIP. Privately insured children, who tend to have higher socioeconomic status, are more likely to bypass their nearest hospital for care and may visit out-of-state hospitals at different rates.^25,26^ However, care use patterns between Medicaid and privately insured children do not substantially differ in aggregate.^27^ Second, some zip codes were large, and we did not know patients’ precise locations within zip codes, nor could we know whether patients actually resided in those zip codes at the time they sought emergent care. We believe such a discrepancy would be uncommon and would generally increase noise in the data. However, the strong fit, as suggested by low confidence intervals, between distance and border crossing in our model suggests that our ability to evaluate out-of-state care was not impaired. Third, telehealth options during our study period may have changed typical ED use, and this may have been associated with the propensity for patients to seek out-of-state care. Fourth, drive times may better inform family decisions for care, although these would be highly correlated with the straight-line distances we used. Fifth, we were unable to evaluate the reasons for seeking out-of-state vs in-state care, such as specialty care. In addition, we could not capture international border-crossing, as may occur into Mexico or Canada.

## Conclusions

In this cross-sectional study, among children across the US enrolled in Medicaid or CHIP, those living near state borders were more likely to access care out of state. Researchers and policymakers should take our findings into account as changes to Medicaid reimbursement could affect patients’ ability to access cross-border care.

## Notes

Supplement 1. **eTable.** Proportion and 95% confidence interval (CI) of patients by state who sought care from another state, or who sought care in another state

Supplement 2.
Data Sharing Statement
