# Supplementary material for: Out-of-State Acute Care Use Among Pediatric Medicaid Enrollees
Source: JAMA Netw Open. 2025 Oct 7;8(10):e2536236. doi: 10.1001/jamanetworkopen.2025.36236 (PMC12505171; doi:10.1001/jamanetworkopen.2025.36236)
Supplement: Supplement 1. — eTable. Proportion and 95% confidence interval (CI) of patients by state who sought care from another state, or who sought care in another state [file jamanetwopen-e2536236-s001.pdf]

Supplementary Online Content

Michelson KA, Singamsetty N, Skol AD, et al. Out-of-state acute care use among pediatric Medicaid enrollees. *JAMA Netw Open*. 2025;8(10):e2536236.
doi:10.1001/jamanetworkopen.2025.36236

eTable. Proportion and 95% confidence interval (CI) of patients by state who sought care from another state, or who sought care in another state

This supplementary material has been provided by the authors to give readers additional information about their work.

© 2025 Michelson KA et al. *JAMA Network Open*.

eTable. Proportion and 95% confidence interval (CI) of patients by state who sought care from another state, or who sought care in another state

State	Left state for care % (95% CI)	Entered state for care % (95% CI)
Alabama	3.6% (3.6-3.7)	2.3% (2.2-2.3)
Alaska	1.6% (1.5-1.7)	1.2% (1.1-1.3)
Arizona	1.4% (1.4-1.5)	1.1% (1.0-1.1)
Arkansas	5.5% (5.4-5.6)	4.2% (4.2-4.3)
California	0.7% (0.7-0.7)	0.2% (0.2-0.2)
Colorado	0.9% (0.9-0.9)	2.8% (2.8-2.9)
Connecticut	1.1% (1.1-1.2)	2.8% (2.8-2.9)
Delaware	3.9% (3.8-4.0)	21.4% (21.2-21.7)
District of Columbia	5.7% (5.5-5.9)	43.7% (43.4-44.0)
Florida	0.8% (0.8-0.8)	1.9% (1.9-1.9)
Georgia	3.5% (3.5-3.5)	2.6% (2.6-2.7)
Hawaii	1.2% (1.2-1.3)	1.1% (1.0-1.1)
Idaho	5.3% (5.2-5.5)	4.3% (4.2-4.4)
Illinois	6.4% (6.4-6.5)	1.3% (1.3-1.3)
Indiana	3.1% (3.1-3.2)	3.5% (3.4-3.5)
Iowa	4.3% (4.2-4.4)	5.4% (5.4-5.5)
Kansas	10.6% (10.4-10.7)	14.3% (14.1-14.4)
Kentucky	5.8% (5.7-5.9)	4.2% (4.2-4.3)
Louisiana	1.8% (1.7-1.8)	2.2% (2.2-2.2)
Maine	2.9% (2.8-3.0)	1.2% (1.1-1.3)
Maryland	15.8% (15.7-15.9)	2.6% (2.6-2.7)
Massachusetts	2.5% (2.4-2.5)	1.0% (1.0-1.0)
Michigan	1.5% (1.5-1.5)	0.6% (0.6-0.6)
Minnesota	3.9% (3.9-4.0)	1.9% (1.8-1.9)
Mississippi	6.3% (6.2-6.4)	5.5% (5.4-5.6)
Missouri	5.7% (5.6-5.7)	7.7% (7.6-7.7)
Montana	2.6% (2.5-2.7)	2.3% (2.2-2.4)
Nebraska	3.8% (3.7-3.9)	5.1% (5.0-5.3)
Nevada	2.0% (2.0-2.1)	3.5% (3.4-3.6)
New Hampshire	6.1% (5.9-6.3)	10.3% (10.1-10.5)
New Jersey	3.6% (3.6-3.7)	3.0% (3.0-3.1)
New Mexico	10.6% (10.4-10.7)	1.5% (1.5-1.6)
New York	2.6% (2.5-2.6)	0.6% (0.5-0.6)
North Carolina	1.9% (1.9-1.9)	3.5% (3.5-3.6)
North Dakota	3.9% (3.7-4.1)	17.0% (16.6-17.3)

© 2025 Michelson KA et al. *JAMA Network Open.*

State	Left state for care<br>% (95% CI)	Entered state for care<br>% (95% CI)
Ohio	2.0% (1.9-2.0)	3.2% (3.2-3.3)
Oklahoma	3.7% (3.6-3.7)	0.6% (0.6-0.7)
Oregon	2.8% (2.8-2.9)	5.5% (5.4-5.6)
Pennsylvania	4.0% (3.9-4.0)	3.3% (3.3-3.4)
Puerto Rico	0.6% (0.5-0.6)	0.3% (0.3-0.3)
Rhode Island	10.2% (9.4-11.0)	62.7% (61.9-63.5)
South Carolina	7.0% (6.9-7.0)	2.5% (2.5-2.5)
South Dakota	4.2% (4.0-4.4)	5.5% (5.3-5.7)
Tennessee	1.2% (1.2-1.3)	7.1% (7.0-7.1)
Texas	0.8% (0.7-0.8)	1.3% (1.3-1.3)
US Virgin Islands	2.2% (1.8-2.6)	0.6% (0.4-0.9)
Utah	7.9% (7.4-8.5)	32.5% (31.7-33.2)
Vermont	11.7% (11.3-12.0)	10.4% (10.1-10.8)
Virginia	4.1% (4.0-4.1)	2.7% (2.7-2.7)
Washington	3.2% (3.1-3.2)	2.4% (2.4-2.5)
West Virginia	11.0% (10.8-11.1)	14.1% (13.9-14.3)
Wisconsin	1.8% (1.7-1.8)	3.4% (3.4-3.5)
Wyoming	8.1% (7.8-8.4)	5.2% (4.9-5.4)

© 2025 Michelson KA et al. *JAMA Network Open*.
