# Supplementary material for: Out-of-State Acute Care Use Among Pediatric Medicaid Enrollees
Source: JAMA Netw Open. 2025 Oct 7;8(10):e2536236. doi: 10.1001/jamanetworkopen.2025.36236 (PMC12505171; doi:10.1001/jamanetworkopen.2025.36236)
Supplement: Supplement 2. — Data Sharing Statement [file jamanetwopen-e2536236-s002.pdf]

Data Sharing Statement

Data
Data available: No

Additional Information
Explanation for why data not available: We are unable to share this data due to a data use agreement restriction.
